# Quantitative Historical Change in Bumblebee (*Bombus* spp.) Assemblages of Red Clover Fields

**DOI:** 10.1371/journal.pone.0025172

**Published:** 2011-09-26

**Authors:** Yoko L. Dupont, Christian Damgaard, Vibeke Simonsen

**Affiliations:** 1 Section of Ecology and Genetics, Department of Bioscience, Aarhus University, Aarhus, Denmark; 2 Section of Terrestrial Ecology (NERI), Department of Bioscience, Aarhus University, Silkeborg, Denmark; Trinity College Dublin, Ireland

## Abstract

**Background:**

Flower visiting insects provide a vitally important pollination service for many crops and wild plants. Recent decline of pollinating insects due to anthropogenic modification of habitats and climate, in particular from 1950's onwards, is a major and widespread concern. However, few studies document the extent of declines in species diversity, and no studies have previously quantified local abundance declines. We here make a quantitative assessment of recent historical changes in bumblebee assemblages by comparing contemporary and historical survey data.

**Methodology/Principal Findings:**

We take advantage of detailed, quantitative historical survey data from the 1930's on bumblebee (*Bombus* spp.) abundances and species composition in red clover (*Trifolium pratense*) fields, an important floral resource and an attractant of all bumblebee species. We used the historical survey data as a pre-industrialization baseline, and repeated the same sampling protocol at nearly the same localities at present, hence setting up a historical experiment. We detected historical changes in abundances (bees/m^2^) of both workers (the “pollinatory units”) and queens (effective population size), in addition to species composition. In particular, long-tongued bumblebee species showed consistent and dramatic declines in species richness and abundances throughout the flowering season of red clover, while short-tongued species were largely unaffected. Of 12 *Bombus* species observed in the 1930's, five species were not observed at present. The latter were all long-tongued, late-emerging species.

**Conclusions/Significance:**

Because bumblebees are important pollinators, historical changes in local bumblebee assemblages are expected to severely affect plant reproduction, in particular long-tubed species, which are pollinated by long-tongued bumblebees.

## Introduction

Pollinators play a key role in natural and agricultural ecosystems, providing an important pollination service of wild plants and crops [1]. Serious concerns have been raised of a recent widespread decline of pollinating insects, potentially leading to a subsequent loss of insect-pollinated wild plants [2], [3] and/or substantial loss of agricultural productivity [4]. Although the existence of a global pollination crisis has been questioned [5], in recent years it has become a widespread perception and evidence is accumulating, that both wild and domesticated pollinators are in decline [6], [7]. Most notably, Biesmeijer et al. [8] presented convincing evidence of parallel decreases in species richness of pollinators (bees and hoverflies) and insect-pollinated plants at a national scale in England and the Netherlands. Agricultural intensification and habitat loss are often identified as the main drivers of pollinator decline [6], [9], [10], although pests and pathogens may also be important [11], [12], at least in some species and in certain regions (America) [13].

For bumblebees (*Bombus* Latreille spp.), which are among the most important and best known group of wild pollinators [14], [15], a long-term decline has been documented locally and regionally, in Europe, America and Asia [9], [13], [16], [17], [18]. In particular, local extinctions and decrease in range extension of some species are apparent from approximately the 1950's onwards [10], [17], [19], [20], [21], although population declines vary regionally [7], [17]. Whereas it is widely agreed that there has been a general decline in the diversity of bumblebees, quantitative documentation of historical changes are almost completely lacking to date [8], [17]. Exceptions include a few studies, in which historical declines of bumblebees are documented on the basis of natural history collections [13], [16]. Museum collections can provide important and accurate information regarding species numbers and distributions, however, they more rarely reflect differences in local abundances due to collector bias [20]. Furthermore, change in species composition of bumblebee assemblages was recently assessed from historical survey data [21]. While the latter study report proportional representation of species, to our knowledge, no studies have directly quantified historical changes in density (bees/m^2^), here termed abundance.

To date, no standardized and repeatable historical survey data have been presented, which may serve as a pre-industrialization baseline of pollinator abundances [8], [17]. However, in Scandinavia there is a long tradition of research on breeding of red clover (*Trifolium pratense* L.) [22], an important and widespread crop in Northern Europe before the intensification of agricultural production in the 1950's onwards [16]. Red clover is self-incompatible, and bumblebees, specifically long-tongued species, are the most important pollinating agents [22], [23]. Moreover, red clover is an important forage of bumblebees [22], [24], [25]. In February 1930, a prize was offered by the Royal Danish Academy of Sciences to investigate the importance of *Bombus* spp. in the pollination of red clover and the distribution of bumblebees and their nests in Denmark. The award winner, O. S. Skovgaard subsequently continued the study of bumblebees in red clover fields 1930–1934. In his publication [26], methods were described sufficiently for a similar study to be replicated at present, providing a unique retrospect to pre-industrialization.

The purpose of the current study is to compare present species composition and abundances of bumblebees in red clover fields to that of the study by Skovgaard from the 1930's. We use a similar sampling design to repeat the historical study at present, to make a quantitative assessment of changes in Danish *Bombus* populations in red clover fields in the last 80 years. We here report a significant change in local species composition and abundances of bumblebees. We found a marked decrease in abundances (in some cases even absence) of most long-tongued species at present, while short-tongued species remain unaffected.

## Materials and Methods

### Historical study, past data

**Figure 1:**
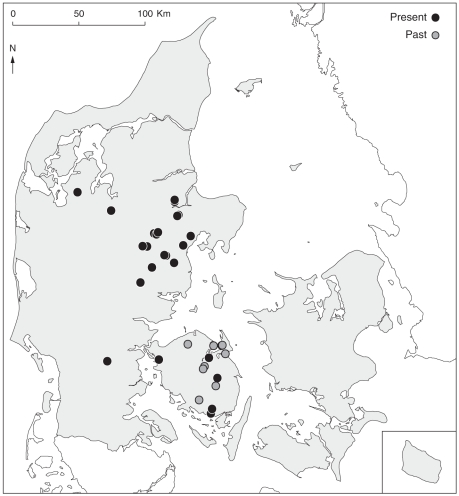
Study sites. Localities of red clover fields in the past [25] (grey) and the present study (black).

Skovgaard monitored nest-building bumblebees (excluding species of sub-genus *Psithyrus*) in a total of 25 red clover fields at 10 localities during five years (1930–34) on the island of Funen, Denmark (Figure 1). Varying numbers of study fields and locations were visited each year (Table 1). Except for three pairs of fields belonging to the same farm, all study fields of a given year were separated by several km. Observations included both early-flowering and late-flowering diploid red clover cultivars of Øtofte and Tystofte. Corolla tube length was approximately 9 mm [26]. Sampling was done from the middle of June until early September, sampling period differing among years (Table 1). Fields were observed before, at and after peak flowering of red clover, but mostly at peak flowering. Abundances of bumblebees peaked mostly in July (mean 23^rd^ of July) for all study fields in all years. All bumblebee observations were done during the daytime (5 to 20 h).

**Table 1:** Sampling effort in past and present field studies.

Year	first day	last day	No fields	No localities	No records^a^
1930	30 June	03 Aug.	3	3	4
1931	11 July	10 Sep.	4	3	9
1932	11 July	12 Aug.	7	7	14
1933	18 July	26 July	6	4	31
1934	17 June	11 July	5	2	12
2008	20 June	06 Aug.	12	12	42
2009	03 June	20 Aug.	17	16	99
Total past			25	10	70
Total present			29	19	141

a For present observations, number of records represents aggregated data (observations aggregated within each observation day for each field).

In 1930–31, study plots were unequal in size, but generally covered a total of 900–1200 m^2^ per field, consisting of 2–3 sub-plots which represented different parts of the field. Sub-plots mostly encompassed six rows across the field, from one edge to the other. In 1932–34, bumblebees were counted in a total of 1000 m^2^ per study field (except one field, in which study plots were only 200 m^2^). No further details are reported on the spatial dimensions and location of the plot(s) within the field, but given Skovgaard's consideration of representing various parts of the field, including edge and center, we assume that several sub-plots were used. Flowering was estimated in the historical study [26] as % flowering (in late season as % withered flowers) or as “peak flowering” only for the first and last day of observation of each study field. Because bumblebee abundances changes through the flowering season of red clover [27], we only included observation days for which flowering estimates were reported in the analysis, *i.e.* 70 sampling records of a total of 119. We arbitrarily classified days of ≤30% flowering as early flowering season, “peak flowering” as mid season, and ≥70% withered as late flowering season. All past data were converted to density (*Bombus* individuals/m^2^) for the analysis.

### Data collection, present data

Relevant permits for conducting field work in red clover fields were obtained. Because red clover is currently a minor crop and fields for seed production rare, we monitored bumblebee abundances in red clover fields in a total of 14 localities in Eastern Jutland, in addition to five localities on Funen, Denmark (Figure 1). The study included nearly all red clover seed fields in the two regions. The study encompassed a total of 29 fields: 12 fields were visited from the 20^th^ of June to the 6^th^ of August 2008, and 17 fields were visited from the 3^rd^ of June to the 20^th^ of August 2009. Fields were separated by at least 10 km, although three pairs of fields were only 1.5–2 km apart. Because most bumblebee species have a foraging range <500 m (although up to 2 km for *B. terrestris*) [28], [29], [30], [31], bumblebee faunas of different fields are expected to be independent. Study fields were all organically grown, a growing practice that more closely imitates agricultural practices of the 1930's (*e.g.* no pesticides and artificial fertilizers, small fields, more insect pollinated crops) than conventional management methods. Furthermore, all fields were grown for seed production, which ensured presence of flowers in the study fields. Currently, only red clover of the early-flowering cultivar, Milvus and the intermediate-late flowering Rajah (both diploid varieties), are used, but flowering time varied among fields due to cutting (which postponed flowering) and soil type (flowering earlier on sandy soils). Average corolla length of the most common cultivar, Rajah, is 8.83 mm [32], while corolla length of Milvus is unknown. Red clover flowered from early June to late August, although in most fields flowering peaked in the middle of July. Each red clover field was observed 2–4 (mean (SD) = 3.1 (0.4)) times during the flowering season of the field, preferably encompassing the beginning, middle and late flowering season.

Bumblebee observations were carried out during the daytime (8 to 19 h) under favorable weather conditions, *i.e.* on days with no rain or strong wind. To mimic the historical study [25], bumblebee counts were carried out in three different sub-plots totaling 1000 m^2^ and representing different spatial parts of the study field. Currently, red clover is broad cast and, hence, it was not possible to select six rows as in the past [25]. The three sub-plots of 18×18 m each were placed on a line, one at the field edge, one at the centre of the field, and one in between. In every sub-plot, the observer walked slowly back and forth in rows, observing each red clover flower head approximately once, and recording the number, caste (workers, queens and males) and species of flower-visiting bumblebees. Workers and queens were distinguished based on size, as was presumably done in the historical study. Parasitic bumblebees (sub-genus *Psithyrus*) were excluded. Only bees alighting on the flower heads and collecting pollen or nectar were registered as flower-visitors. The observer walked through the sub-plots1-4 times on each sampling day, thereby sampling the abundance of bumblebees in 324–1296 m^2^.

On each sampling day, flowering was estimated by counting the number of red clover flower heads in three 1×1 m squares placed diagonally in each of the sub-plots. Because red clover fields flower for a prolonged period, we assumed that each field was visited at least once during peak flowering. Peak flowering was defined as the day of maximum flower head density of the 1×1 m squares (mean (SD)). Flowering of the field was categorized as early (if flowering before mid, and mean (SD) flower head density not overlapping with mid flowering), mid (maximum observed flower head density) or late flowering (if flowering after mid, and mean (SD) flower head density not overlapping with mid flowering).

Sample specimens of bumblebees were collected at all sites and all sampling days for later identification by a taxonomic expert (Henning Bang Madsen, Copenhagen University). Bee species data were classified and aggregated into long-tongued and short-tongued bumblebee species (Table 2). The recorded data from the same field and the same day of the present collections were aggregated, so that both the sampled area of past and present data were of the same order of magnitude and had the same level of aggregation.

**Table 2:** Total numbers of bumblebees observed in the red clover fields in the past and present.

		No workers		No queens	
Functional group	*Bombus* species	past	present	past	present
Long-tongued	*B. hortorum*	1424	858	24	23
	*B. pascuorum*	349	2307	6	19
	*B. muscorum*	122	236	3	0
	*B. distinguendus*	857	0	24	0
	*B. sylvarum*	52	0	2	0
	*B. veteranus*	121	0	5	0
	*B. ruderarius*	21	0	0	0
	*B. subterraneus*	17	0	2	0
Short-tongued	*B. terrestris*	3906	13580	21	349
	*B. lapidarius*	445	3499	12	98
	*B. hypnorum*	17	12	1	0
	*B. pratorum*	29	24	0	0
	Total	7360	20516	100	489

Bumblebee species were classified as long-tongued or short-tongued on the basis of tongue lengths measured in [24]. In both past and present studies, individuals belonging to *B. terrestris* (L.) and the *B. lucorum* complex (*B. lucorum* L., *B. magnus* Vogt. and *B. cryptarum* (F.)) were recorded as one species (hereafter *B. terrestris*). These species are difficult to distinguish in the field, but functionally similar [53], [54]. Notice that the sampling intensity differed between past and present studies, and the observed numbers of bees are, hence, not directly comparable.

### Statistical analysis

The aggregated numbers of observed long- and short-tongued bumblebee workers and queens were divided by the sampled area. Males were numerically rare in both past and present, and omitted from the analyses. Histograms of the area-corrected number of bumblebees showed that the area corrected number of bumblebees (bees/m^2^) was approximately exponentially distributed (Figure S1). Consequently, the change in the area-corrected number of observed long- and short-tongued bumblebees between the past and the present, respectively, were analysed under the assumption that the area corrected number of bees is exponentially distributed with the inverse of the mean area-corrected number of bees as the rate parameter. We used likelihood ratio tests on the rate parameters to test possible differences between the past and the present. Furthermore, we analysed changes in the observed species distribution assuming that the number of observed bee species are multinomial distributed, and differences were tested by the use of likelihood ratio tests on the vector of observation probabilities.

We tested for differences in abundance (bees/m^2^) and species composition (proportional representation of species) of bumblebee groups. Analyses were done for all species, in addition to long-tongued and short-tongued bumblebees and for queens and workers separately. For present data, differences among sub-plots at the edge, mid and centre of the field were tested. Queens were more common at the field edges (long-tongued: χ^2^ = 13.00, *P*<0.001; short-tongued: χ^2^ = 10.95, *P*<0.001), but always numerically rare. Abundances of workers did not differ significantly among sub-plots within a field on a given sampling day (*P*>0.01) (Figure S2). Only minor differences were found in species composition (Table S1). Hence, to enable comparison with past data, present data of sub-plots were aggregated within fields for each observation day. Differences between Jutland and Funen were tested for present data (all past data were from Funen). No regional differences (Jutland *versus* Funen) were found in abundances of bumblebees (*P*>0.01) (Figure S3), and only small differences in species composition (Table S2), justifying a comparison to past data from Funen.

The effect of season (early, mid and late flowering) was tested for both past and present data. Generally, abundance and species composition of bumblebees were significantly affected by flowering season in both present and past. Hence, we tested whether aggregated data differed between past and present in early, mid and late season, respectively.

## Results

### Abundances

**Figure 2:**
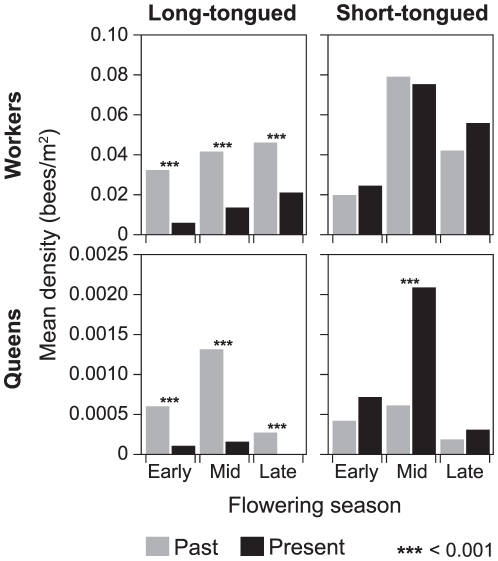
Historical change in bumblebee abundances. Mean bumblebee abundance (bees/m^2^) of long tongued (left) and short tongued (right) workers (top) and queens (bottom) during the beginning, mid and late flowering season of the red clover fields in the past (grey) and at present (black).

We currently observed a total of 20516 workers (16.6% long-tongued) and 489 queens (8.6% long-tongued), while Skovgaard observed 7360 workers (40.3% long-tongued) and 100 queens (66.0% long-tongued) in the past (Table 2). In all three periods during the flowering season of red clover, long-tongued workers and queens showed steep and significant abundance declines from past to present (Figure 2). In contrast, we generally found no significant difference in abundances for short-tongued bumblebees from past to present, and even significant increase in abundance of short-tongued queens in mid season (Figure 2). Overall, no significant historical abundance declines of all bumblebee workers or queens were detected (all *P*>0.01), possibly due to a general rarity of long-tongued species.

### Species composition

**Figure 3:**
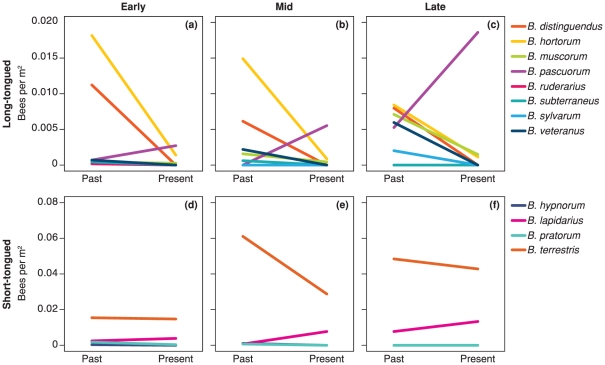
Historical change in bumblebee species composition. Abundances (bees/m^2^) of long-tongued (top) and short-tongued (bottom) species and direction of change from past to present in early, mid and late flowering season of the red clover fields.

Species composition, *i.e.* proportional representation of species, differed significantly between past and present for both long-tongued and short-tongued species throughout the flowering season (all *P*<0.01). Generally, bumblebee assemblages at present had much lower abundances of long-tongued species (Figure 3). Skovgaard repeatedly observed 12 *Bombus* spp. in each study year in the past (13, if including *B. soroeensis*, for which no quantitative data were registered [25]). In contrast, only seven species were observed at present (2008 and 2009) despite a wider geographical range of study sites and a more intensive sampling effort (Table 1 and Figure 3). Five species had disappeared from past to present (*B. distinguendus*, *B. sylvarum*, *B. veteranus*, *B. ruderarius*, *B. subterraneus*). These were all long-tongued, late-emerging species. Only one long-tongued species, *B. pascuorum*, had increased, in particular in mid and late flowering season (Figure 3).

## Discussion

### Historical decline of pollinators: regional and local patterns

It is widely perceived that the regional bumblebee species richness and range extent of some species have declined historically across Europe [9], [10], [16], [18]. However, status and trends of pollinators are based mostly on red list assessments and studies of museum collections, while few quantitative data exist. The current study is one of the first accounts on the basis of historical survey data, which document local population declines in species richness and abundance of some species, but not others. Historically, rare long-tongued species of bumblebees were observed regularly in fields of red clover, albeit in low numbers, in addition to the historically abundant long-tongued *B. distinguendus* (whose common name in Danish is translated as the “Clover Bumblebee”) [26]. At present, only currently widespread and commonly occurring species were observed in the red clover fields. Bumblebee species of low abundance in red clover fields are common elsewhere (*B. hypnorum* in urban areas) or at other times during the season (*B. pratorum* in early summer) [14], [33]. However, none of the historically rare species were registered in the present investigation, nor was the historically common *B. distinguendus* observed, despite intensive sampling across the season at multiple locations. Historical trends in species composition of bumblebees of the current study are similar to that of a recent Swedish study, although some of the rare long-tongued species were registered at present in Sweden [21]. The agricultural landscape in Denmark, which is more intensively farmed, appears to be even more depauperate in long-tongued bumblebees, including *B. hortorum*, which is common in other habitats, *e.g.* gardens [33]. One exception to this pattern is the moderate to long-tongued generalist *B. pascuorum*, which has increased historically. Overall, for bumblebee communities of red clover fields of the current study, abundances in addition to species richness of long-tongued species have declined dramatically, while short-tongued species are largely unaffected. For long-tongued species, abundances of queens was found to decline an order of magnitude from the 1930's to the present, corresponding to a dramatic decline in effective population sizes. Findings of the present study are strong and direct evidence of local changes in species richness and abundances, which largely corroborate existing knowledge of larger scale regional changes of bumblebee species occurrences [15].

### Possible causes of decline

Red clover is attractive for all bumblebee species, including rare and declining species [22], [24], [25], and hence the bumblebee assemblages observed in red clover fields is expected to be a good indicator of the regional species pool of bumblebees. The decline of some species of bumblebees and absence of others indicate that requirements of these species are not met at present in the modern agricultural landscape. There is a general agreement among bumblebee ecologists that historical changes in agricultural practices and land use are consistent drivers of bumblebee decline since the industrialization [18]. Adverse effects of historical changes in agricultural practices and land use include increased mortality due to pesticide application [34], pathogen spillover [11] and possible competition [27] from commercial bees, changes in landscape configuration leading to loss of hibernation and nesting sites [35], [36], [37], reduced sowing of leguminous crops (including red clover) and flower-rich meadows [16], [38], in addition to gaps in continuity of floral resources throughout colony life [30].

However, why some species have declined while others remain abundant is a long-standing question (*e.g.* [18], [24], [39], [40]). The most prevalent hypotheses include (1) diet specialization and decline of preferred food plants [9], [16], [24], [41] and (2) small geographical range size, reflecting a narrow climatic niche constrained by physiological tolerances [18], [39]. For both hypotheses, specificity is a key element, implying that specialized species are predisposed to decline and extirpation. Concordant with this pattern, the short-tongued species of the current study are all opportunistic in choice of food plants [26] and nesting sites [26], [37], while little is known about hibernation sites [35]. Furthermore, the by far most dominant bumblebees in red clover fields, the short-tongued *Bombus terrestris* and *B. lucorum* complex, have a relatively large foraging range, some studies reporting up to 1.5–2 km [30], [31]. Thus, these species may respond to the environment at larger spatial scales [42], and may be less vulnerable to *e.g.* habitat fragmentation and loss. In contrast, known foraging distances of other *Bombus* spp., including the long-tongued *B. pascuorum*, *B. muscorum* and *B. hortorum*, are limited to a few hundred meters [29], [31], [43].

Long-tongued bumblebee species have a strong preference for long-tubed flowers, including red clover [24], [38]. One hypothesis is that the historical decline of long-tongued bumblebees is linked to a general decline in red clover fields and other leguminous crops [16], [38], [44]. However, the rare and declining species *B. veteranus*, *B. distinguendus* and *B. sylvarum* are currently found in Denmark only at coastal meadows, which are highly diverse in plants and insects, but not specifically rich in red clover [45]. Thus, whereas the decline in availability of red clover fields may contribute to decline of long-tongued species, other factors are likely to be involved. For instance, the declining species also tend to emerge late in the season compared to stable species (this study, [40]). We lack knowledge about interspecific variation in habitat use and vulnerability during critical stages of the life cycle, including nest initiation, colony build-up and climax, reproduction and hibernation [14], [15]. Moreover, drivers of historical changes are difficult to assess because decline itself may influence observed patterns, *e.g.* present floral specialization may reflect absence or rarity of forage plants [18], [39]. Here, historical survey data may provide a reliable picture of the past.

### Shifts in bumblebee functional groups and consequences for pollination

Bumblebees are, perhaps, the most important group of wild pollinators in Northern Europe, due to their ability to forage at low temperatures [46], capability of buzz pollination [47] and ability to handle complex flowers [48], their relatively long tongues compared to other bee species [14], [26], and generally broad floral diet despite preferences [39]. The changes in species composition and abundances of bumblebee workers in red clover fields result in a marked shift in composition of functional groups of bumblebees towards lower abundances of long-tongued bumblebees and stable abundances of short tongued species. Queens of the short-tongued *Bombus terrestris* have increased in mid season, perhaps due to niche space vacated by the dramatic decline of long-tongued bumblebee queens. Colonies of *Bombus terrestris* are among the most highly populated [19], which may further elevate the proportional representation of short-tongued workers. Because workers account for the vast majority of flower-visits by bumblebees [14], the compositional change of bumblebees may have severe implications for the pollination environment of plants [1]. At present, however, no historical abundance change of *B. terrestris* was observed.

Long-tongued bumblebees are important pollinators of several crops, including clovers (*Trifolium* spp.), field bean (*Vicia faba*), and a range of berries, fruits and vegetables [22]. Long-tongued species prefer to visit long-tubed flowers [49]. For red clover, which has the longest floral tube among Northern European plants, short-tongued bumblebees are not optimal pollinators and regularly act as non-pollinating nectar robbers [22], [26]. Decreasing seed yields have been reported, rendering red clover a crop of low profit [[21] (Sweden), C. Jørgensen and S. Oddershede, pers. com. (Denmark)].

Many wild plants are visited and pollinated predominantly or exclusively by bumblebees [15]. In plant-flower-visitor networks, which encompasses all co-occurring species of plants and visitors, bumblebees are suggested as important hubs [3], [50] or generalist core species [51], [52], which act as keystone pollinators in the system. Hence, species extinctions, local decline and shifts in functional group composition of bumblebees are expected to heavily impact the structural organization and functioning of natural pollination networks.

Results of the current study, thus, are strong direct evidence of extensive historical changes in abundance and species composition of local bumblebee populations in Denmark, changes, which have previously been assumed but scarcely documented. Although historical studies are highly variable in scientific quality, the use of appropriate historical survey data repeated at present, shows great potential for retrospective analyses, and hence “a look into the past”.

## Supporting Information

Figure S1 **Distributions of observed and expected abundances of bumblebees.** Histograms of observed abundances of bumblebees (m^−2^) in mid season compared to the expected abundances under the assumption that data are exponential distributed. The same plots for early and late seasons were qualitatively similar.
(EPS)

Figure S2 **Within-field differences in bumblebee abundances at present.** Spatial differences in bumblebee abundances in sub-plots at the edge (light grey) middle (dark grey) and center (black) within fields. Likelihood ratio test of the effect of subplot: ** *P*<0.001.
(EPS)

Figure S3 **Regional differences in bumblebee abundances at present.** Regional differences in bumblebee abundances between Jutland (grey) and Funen (black). Likelihood ratio test of the effect of subplot: ** *P*<0.001.
(EPS)

Table S1 **Within-field differences in species composition of bumblebee assemblages at present.** Total numbers of bumblebees observed in sub-plots at the edge, middle and center of the red clover fields in the present study.
(DOC)

Table S2 **Regional differences in species composition of bumblebee assemblages at present.** Total numbers of bumblebees observed in Jutland and Funen in the present study.
(DOC)
