# Supplementary material for: Quantitative Historical Change in Bumblebee (Bombus spp.) Assemblages of Red Clover Fields
Source: PLoS One. 2011 Sep 26;6(9):e25172. doi: 10.1371/journal.pone.0025172 (PMC3180388; doi:10.1371/journal.pone.0025172)
Supplement: Table S1 — Within-field differences in species composition of bumblebee assemblages at present. Total numbers of bumblebees observed in sub-plots at the edge, middle and center of the red clover fields in the present study. (DOC) [file pone.0025172.s004.doc]

*Table S1. Within-field differences in species composition of bumblebee assemblages at present.*

|  |  | Workers^a^ | | |  | Queens^b^ | | |
| --- | --- | --- | --- | --- | --- | --- | --- | --- |
| Functional group | *Bombus* species | Edge | Middle | Central |  | Edge | Middle | Central |
| Long-tongued | *B. hortorum* | 247 (3.7%) | 261 (3.7%) | 350 (5.0%) |  | 7 (3.8%) | 9 (5.4%) | 7 (4.7%) |
|  | *B. pascuorum* | 920 (13.6%) | 742 (10.5%) | 645 (9.2%) |  | 9 (4.9%) | 6 (3.6%) | 4 (2.7%) |
|  | *B. muscorum* | 58 (0.9%) | 94 (1.3%) | 84 (1.2%) |  | 0 | 0 | 0 |
|  | *B. distinguendus* | 0 | 0 | 0 |  | 0 | 0 | 0 |
|  | *B. sylvarum* | 0 | 0 | 0 |  | 0 | 0 | 0 |
|  | *B. veteranus* | 0 | 0 | 0 |  | 0 | 0 | 0 |
|  | *B. ruderarius* | 0 | 0 | 0 |  | 0 | 0 | 0 |
|  | *B. subterraneus* | 0 | 0 | 0 |  | 0 | 0 | 0 |
| Short-tongued | *B. terrestris* | 4318 (63.9%) | 4784 (67.8%) | 4706 (67.4%) |  | 140 (75.7%) | 116 (69.5%) | 104 (70.3%) |
|  | *B. lapidarius* | 1194 (17.7%) | 1161 (16.5%) | 1189 (17.0%) |  | 29 (15.7%) | 36 (21.6%) | 33 (22.3%) |
|  | *B. hypnorum* | 6 (0.1%) | 4 (0.1%) | 4 (0.1%) |  | 0 | 0 | 0 |
|  | *B. pratorum* | 12 (0.2%) | 5 (0.1%) | 7 (0.1%) |  | 0 | 0 | 0 |
|  | Total | 6755 (100%) | 7051 (100%) | 6985 (100%) |  | 185 (100%) | 167 (100%) | 148 (100%) |

Total numbers of bumblebees (% of total) observed in sub-plots at the edge, middle and center of the red clover fields in the present study. Notice that the sampling intensity did not differ between sub-plots at the edge, middle and center, and the observed numbers of bees are, hence, comparable.

^a^ Significant spatial differences in species composition was found only for long-tongued workers (χ^2^ = 69.29, *P* *<* 0.001) primarily due to a lower density of *Bombus pascuorum* towards the center of the fields

^b^ No differences in species composition was found among sub-plots within fields (*P >* 0.01)
