# Supplementary material for: Quantitative Historical Change in Bumblebee (Bombus spp.) Assemblages of Red Clover Fields
Source: PLoS One. 2011 Sep 26;6(9):e25172. doi: 10.1371/journal.pone.0025172 (PMC3180388; doi:10.1371/journal.pone.0025172)
Supplement: Table S2 — Regional differences in species composition of bumblebee assemblages at present. Total numbers of bumblebees observed in Jutland and Funen in the present study. (DOC) [file pone.0025172.s005.doc]

*Table S2. Regional differences in species composition of bumblebee assemblages at present.*

|  |  | Workers^a^ | |  | Queens^b^ | |
| --- | --- | --- | --- | --- | --- | --- |
| Functional group | *Bombus* species | Jutland | Funen |  | Jutland | Funen |
| Long-tongued | *B. hortorum* | 605 (3.9%) | 253 (4.9%) |  | 13 (3.4%) | 10 (8.9%) |
|  | *B. pascuorum* | 1677 (10.8%) | 630 (12.1%) |  | 17 (4.4%) | 2 (1.8%) |
|  | *B. muscorum* | 179 (1.1%) | 57 (1.1%) |  | 0 | 0 |
|  | *B. distinguendus* | 0 | 0 |  | 0 | 0 |
|  | *B. sylvarum* | 0 | 0 |  | 0 | 0 |
|  | *B. veteranus* | 0 | 0 |  | 0 | 0 |
|  | *B. ruderarius* | 0 | 0 |  | 0 | 0 |
|  | *B. subterraneus* | 0 | 0 |  | 0 | 0 |
| Short-tongued | *B. terrestris* | 10624 (68.2%) | 3184 (61.1%) |  | 274 (70.6%) | 86 (76.8%) |
|  | *B. lapidarius* | 2457 (15.8%) | 1087 (20.8%) |  | 84 (21.6%) | 14 (12.5%) |
|  | *B. hypnorum* | 14 (0.1%) | 0 |  | 0 | 0 |
|  | *B. pratorum* | 20 (0.1%) | 4 (0.1%) |  | 0 | 0 |
| Total |  | 15576 (100%) | 5215 (100%) |  | 388 (100%) | 112 (100%) |

Total numbers of bumblebees (% of total) observed in Jutland and Funen in the present study. Notice that the sampling intensity differed between the two regions, and the observed numbers of bees are, hence, not directly comparable.

^a^ Significant regional differences in species composition was found only for short-tongued workers (χ^2^ = 93.75, *P* *<* 0.001)

^b^ No regional differences (*P* > 0.01)
